# Experimental Investigation of the Effect of Purge Flow and Main Flow Interaction in a Low-Speed Turbine Cascade Passage

**DOI:** 10.3390/e22060623

**Published:** 2020-06-04

**Authors:** Lianpeng Zhao, Hongwei Ma

**Affiliations:** School of Energy and Power Engineering, Beihang University, Beijing 100191, China; zlpbuaa@buaa.edu.cn

**Keywords:** turbine cascade, purge flow, PIV, interaction, vortex identification, Rortex, passage vortex, mean entropy production

## Abstract

In order to protect the vulnerable turbine components from extreme high temperature, coolant flow is introduced from the compressor to the disk cavity, inevitably interacting with the main flow. This paper describes an experimental investigation of the interaction between the main flow and the purge flow in a low-speed turbine cascade with three purge flow rates, Cm = 0, Cm = 1%, and Cm = 2%. In order to study the effect of the interaction between the main flow and the purge flow on the secondary flows, a Rortex method developed by Liu Chaoquan is introduced to identify the vortex in the flow field. In the meantime, a method to calculate the mean entropy production rate based on the particle image velocimetry (PIV) result is adopted to investigate the flow loss. The PIV result indicates that the purge flow has a prominent impact on the flow field of the cascade passage, changing the velocity distribution that induces a local blockage area. The results of vortex identification show that the purge flow promotes the generation of the passage vortex near the suction side. In addition, the purge flow makes the passage vortex migrate to the tip wall direction, enlarging the region affected by the secondary flow. The mean entropy production (MEP) result shows that the flow loss is mainly caused by the passage vortex. The coincidence of the high-MEP region and the location of the passage vortex indicates that the purge flow increases the secondary flow loss by affecting the formation and the migration of the passage vortex.

## 1. Introduction

With the demand of higher thermal efficiency, the inlet temperature of modern gas turbines has been increasing over the past decades, intensifying hot gas ingestion. In order to protect the vulnerable turbine components from hot gas ingestions, coolant air from the high-pressure compressor is introduced to the disk cavity between the rotor and stator. However, excess purge flow has a negative effect on the turbine as the purge flow does not work on the turbine blades. Furthermore, the purge flow will interact with the main flow, causing secondary flow loss. In the last few decades, there has been a number of studies on the hot gas ingestion and the interaction of the main flow with the purge flow.

Early studies mainly focused on the hot gas ingestion induced by rotation of the rotor disk. Bayley and Owen, etc. [1] described the experiment of an air-cooled gas turbine using the model of a disk rotating near a shrouded stator, and they developed an experiential formula to predict minimum mass flow to prevent hot gas ingestion. Further studies were carried out by Phadke and Owen [2,3,4,5] using flow visualization, pressure, and concentration measurements to determine C_w,min_ for seven different seal geometries in a wide range of rotational Reynolds numbers up to Re_ɵ_ = 1.2 × 10^6^ and external-flow Reynolds numbers up to Re _w_ = 1.2 × 10^6^. Chew, etc. [6,7] investigated the rim sealing of rotor-stator wheel spaces with and without external flow. They present experimental measurements of the minimum flow rate required to prevent ingestion into a shrouded disc system for three different types of seals, and their results agreed well with former studies. 

Green et al. [8] first investigated the effect of Nozzle Guide Vanes (NGV) and rotor blades on the hot gas ingestion. However, they found that the mainstream ingestion seal flow rates were significantly reduced by the effect of NGV and the rotor blades. Experiments and numerical simulations carried out by Green et al. [9] indicated that the hot gas ingestions could not be completely prevented with circumferential pressure gradients caused by the guide nozzles. Later, Bohn et al. [10] found that the rotor caused a considerable drop in sealing efficiency for a simple axis rim seal, while for the radial rim seal, the result was completely the opposite. Owen [11,12] indicated that there were three kinds of hot gas ingestions according to their origins, the externally induced (EI) ingress, the rotationally induced (RI) ingress, and the combined ingress (CI). This method of classification was widely used by the following researchers [13,14,15].

One of the first studies on the interaction of purge flow and main flow was carried out by McLean et al. [16,17], and the loss coefficient, velocity field, exit angle, and total-to-total efficiency were found to be greatly influenced by the interactions. Schuepbach et al. [18] found that the injection of fluid caused a decrease in the turbine efficiency and a different and generally more unsteady flow field at the rotor exit near the hub. An experimental investigation based on a high-accuracy five-hole probe performed by Regina et al. [19] showed that the purge flow had a negative effect on the turbine efficiency. Furthermore, in order to reduce the secondary loss caused by the purge flow, profiled end walls [20,21,22] were used to control the main flow.

Therefore, present experimental studies mainly focus on the inter-stage flow field or the stage efficiency affected by the interaction of main flow and purge flow, leaving the flow within the turbine passage not fully understood. In addition, more experimental data are needed to validate and support the numerical simulations. In the current paper, the flow field within a turbine cascade passage with three purge flow rates is studied with particle image velocimetry (PIV). To the author’s knowledge, it is the first time that a stereoscopic PIV technique is adopted to investigate the interaction of the main flow and purge flow within the turbine cascade passage.

## 2. Methodology 

### 2.1. Experimental Methods

**Figure 1:**
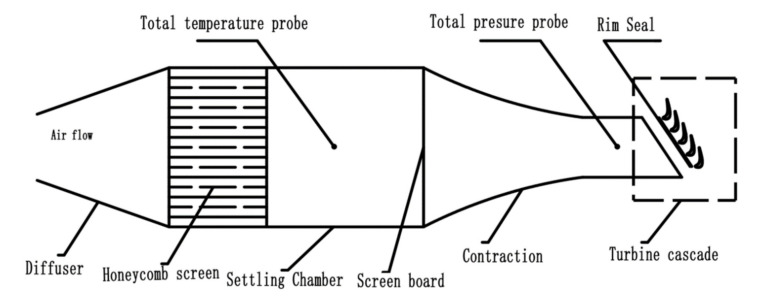
Low-speed wind tunnel.

The experiment was carried out on a turbine cascade in a low-speed wind tunnel, as shown in Figure 1. The rotor blade profile is obtained from a cross-section of a three-dimensional blade in a large-sized gas turbine test equipment. The parameters of the blade are listed in Table 1. A slot size of 3 mm × 375 mm (width × length) was set 25 mm upstream of the blade leading edge to simulate the rim seal of gas turbines, and the purge flow was generated by a centrifugal compressor with a pressure head of 40 kPa. The purge flow rate was measured by a Venturi tube and controlled by an AC drive. In order to ensure the uniformity of the purge flow, a diffuser and a cavity were arranged to connect the pipeline with the rim seal. The inlet velocity of the cascade was set to 15 m/s. The inlet boundary layer was measured as it directly interacts with the purge flow. The thickness of the boundary layer on the hub wall was about 3 mm.

A stereoscopic PIV system, developed by the MicroVec Incorporation, was adopted to measure the velocity field of the cascade passage. The PIV system includes a double-pulse Nd:YAG (neodymium-doped yttrium aluminum garnet; Nd:Y_3_Al_5_O_12_) laser generator with a 532 nm wavelength and a maximum pulse energy of 200 mJ, two CCD cameras with a resolution of 2072 × 2072 pixels, and a liquid drop generator that can generate 1 μm-diameter flow-tracing particles. 

**Figure 2:**
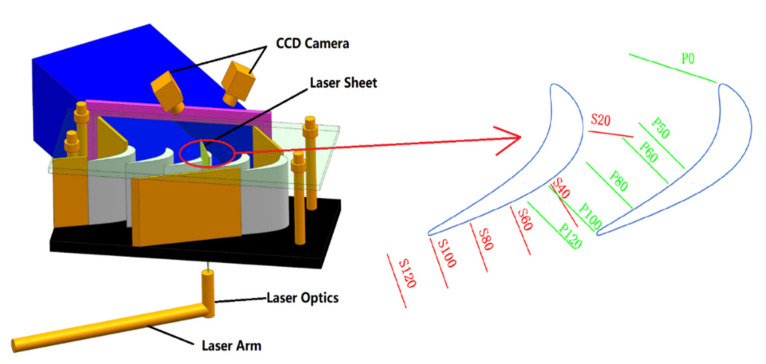
Measurement arrangements.

The arrangement of equipment and location of the measurement sections are shown in Figure 2. The laser optics is installed under the cascade to eliminate the reflection of the hub wall. Furthermore, an anti-reflection film is used to improve the transparency of the hub wall. Six measurement planes are arranged near the suction side (red mark) and the pressure side (green mark). Half of the span from the hub wall is measured to ensure the spatial resolution. Some of the measurement planes are not perpendicular to the blade midline, as the rotor blades obstruct the view of the CCD cameras. As the sealing flow in aircraft engines is about 1% to 2% of the main flow, three cases with a purge flow rate of 0, 1%, and 2% of the main flow are investigated. One thousand pairs of snapshots are acquired at a sampling rate of 10 Hz for each case. The result of each pair of images is calculated by the software Micro Vec V3. The uncertainty of the instantaneous PIV results is about 3.5% of the maximum velocity. For more information about the uncertainty, one can refer to [23,24], in which the same PIV system was adopted.

### 2.2. Entropy Production in Turbulent Flows

The entropy transport equation can be written as [25]

(1) $$\dot{{\dot{P}}_{s}=\frac{k}{T^{2}}}{\left(\frac{\partial T}{\partial x_{i}}\right)}^{2}+\frac{\tau_{ij}}{T}\frac{\partial u_{j}}{\partial x_{j}},$$

where ${\dot{P}}_{s}$ represents the entropy production rate, k is the thermal conductivity, and $\tau_{ij}$ is the viscous stress arising from velocity gradients in the fluid motion. For Newtonian fluid, $\tau_{ij}$ can be expressed as

(2) $$\tau_{ij}=\mu \left[\left(\frac{\partial u_{i}}{\partial x_{j}}+\frac{\partial u_{j}}{\partial x_{i}}\right)-\frac{2}{3}\frac{\partial u_{k}}{\partial x_{k}}\delta_{ij}\right],$$

where μ is the dynamic viscosity and $\delta_{ij}$ is the Kronecker delta. The last term of Equation (2) equates to zero under the assumption of flow incompressibility. For turbulent flow, subdividing entropy into mean and fluctuating components and substituting $\frac{\partial \left(lnT\right)}{\partial x_{i}}$ for $\frac{1}{T}\frac{\partial \left(T\right)}{\partial x_{i}}$, the following result can be obtained [26]:

(3) $$\bar{{\dot{P}}_{s}}=k\frac{\partial }{\partial x_{i}}\left(\bar{\ln T}\right)\frac{\partial }{\partial x_{i}}\left(\bar{\ln T}\right)+k\bar{\frac{\partial }{\partial x_{i}}\left(lnT\right)\frac{\partial }{\partial x_{i}}\left(lnT\right)}+\mu \left[\bar{\frac{1}{T}}\left(\left(\frac{\partial \bar{u_{i}}}{\partial x_{j}}+\frac{\partial \bar{u_{j}}}{\partial x_{i}}\right)\frac{\partial \bar{u_{i}}}{\partial x_{j}}\right)\right]+\;\mu \left[\bar{\frac{1}{T}}\left(\bar{\left(\frac{\partial u_{i}{}^{'}}{\partial x_{j}}+\frac{\partial u_{j}{}^{'}}{\partial x_{i}}\right)\frac{\partial u_{i}{}^{'}}{\partial x_{j}}}\right)\right]+2\mu \frac{\partial \bar{u_{i}}}{\partial x_{j}}\;\bar{{\left(\frac{1}{T}\right)}^{'}\frac{\partial u_{i}{}^{'}}{\partial x_{j}}}+\mu \frac{\partial \bar{u_{i}}}{\partial x_{j}}\;\bar{{\left(\frac{1}{T}\right)}^{'}\frac{\partial u_{j}{}^{'}}{\partial x_{i}}}+\mu \frac{\partial \bar{u_{j}}}{\partial x_{i}}\;\bar{{\left(\frac{1}{T}\right)}^{'}\frac{\partial u_{i}{}^{'}}{\partial x_{j}}}+\;\mu \left[\left(\bar{{\left(\frac{1}{T}\right)}^{'}\left(\frac{\partial u_{i}{}^{'}}{\partial x_{j}}+\frac{\partial u_{j}{}^{'}}{\partial x_{i}}\right)\frac{\partial u_{i}{}^{'}}{\partial x_{j}}}\right)\right],$$

where the overbar and prime notations refer to mean and fluctuating components associated with the Reynolds averaging, respectively. The first two terms of Equation (3) are entropy production terms caused by thermal fluctuations and transport, respectively. The third term represents entropy production due to mean viscous effects. The fourth term represents entropy production caused by dissipation of turbulent kinetic energy. The fifth to seventh terms are the conservation of entropy production, due to mean viscous effects, irreversibilities of fluctuating viscous-temperature effects, and back, respectively. The last term represents the entropy production by the interaction of fluctuating viscous effects and temperature fluctuations. For the 2D flow, ignoring the effect of temperature fluctuations and the temperature gradients, Equation (3) can be expressed as

(4) $$\bar{{\dot{P}}_{s}}=\frac{2\mu }{T}\left[{\left(\bar{\frac{\partial u}{\partial x}}\right)}^{2}+{\left(\bar{\frac{\partial v}{\partial y}}\right)}^{2}\right]+\frac{\mu }{T}{\left[\left(\bar{\frac{\partial v}{\partial x}}\right)+\left(\bar{\frac{\partial u}{\partial y}}\right)\right]}^{2}+\frac{2\mu }{T}\bar{\left[{\left(\frac{\partial u^{\prime }}{\partial x}\right)}^{2}+{\left(\frac{\partial v^{\prime }}{\partial y}\right)}^{2}\right]}+\frac{\mu }{T}\bar{\left(\frac{\partial v^{\prime }}{\partial x}\right)+{\left(\frac{\partial u^{\prime }}{\partial y}\right)}^{2}},$$

In this paper, u and v represent the velocity components within the measurement plane, while w is the velocity component perpendicular to the measurement plane.

### 2.3. Vortex Identification with Rortex Method

The vortex is one of the typical structures in turbulent flows, especially in turbomachines. In the last decades, a number of vortex identification methods have been proposed, such as Q criterion, λ_2_ criterion, and λ_ci_ criterion. However, these traditional methods cannot identify the swirl axis or orientation, nor distinguish the effect of shearing. Liu, etc. [27,28] developed a Rortex method that decomposes the vorticity vector into a rigidly rotational vector and a non-rotational vector. 

In the Rortex method, the local rotation axis Z at point P is defined as a local axis relative to which the fluid has rotational motion only in the plane orthogonal to the Z axis. The XY plane orthogonal to the Z axis is defined as the local rotational plane.

Consider a point P in a reference frame xyz and assume that the local rotation axis of the point P is a vector $r=r_{x}i+r_{y}j+r_{z}k$. Through real Schur decomposition, it can be proved that the reference frame xyz can be transformed to a new reference frame XYZ in which the fluid-rotational axis vector **r** is parallel to the axis Z. The velocity gradient tensor ∇**v** and ∇**V** in reference frames xyz and XYZ, respectively, can be expressed as follows:

(5) $$\nabla v=\left[\begin{array}{ccc} \frac{\partial u}{\partial x} & \frac{\partial u}{\partial y} & \frac{\partial u}{\partial z}\\ \frac{\partial v}{\partial x} & \frac{\partial v}{\partial y} & \frac{\partial v}{\partial z}\\ \frac{\partial w}{\partial x} & \frac{\partial w}{\partial y} & \frac{\partial w}{\partial z} \end{array}\right]\;\;\;\nabla V=\left[\begin{array}{ccc} \frac{\partial U}{\partial X} & \frac{\partial U}{\partial Y} & 0\\ \frac{\partial V}{\partial X} & \frac{\partial V}{\partial Y} & 0\\ \frac{\partial W}{\partial X} & \frac{\partial W}{\partial Y} & \frac{\partial W}{\partial Z} \end{array}\right],$$

The relationship between the two coordinates is

(6) $$\nabla V=Q\nabla vQ^{-1},$$

The rotation axis r is

(7) $$r=Q^{T}\left[\begin{array}{c} 0\\ 0\\ 1 \end{array}\right],$$

When frame XYZ rotates around the Z-axis, the rotation strength should be min(|$\frac{\partial U}{\partial Y}$|,|$\frac{\partial V}{\partial X}$|), which represents the rigid rotation without any deformation. Assuming that frame XYZ rotates around the Z-axis by angle θ, there are

(8) $$\frac{\partial V}{\partial X}{|}_{\theta }=\alpha \sin\left(2\theta +\varphi \right)+\beta ,$$

(9) $$\frac{\partial U}{\partial Y}{|}_{\theta }=\alpha \sin\left(2\theta +\varphi \right)-\beta ,$$

where

(10) $$\alpha =\frac{1}{2}\sqrt{{\left(\frac{\partial V}{\partial X}-\frac{\partial U}{\partial Y}\right)}^{2}+{\left(\frac{\partial V}{\partial X}+\frac{\partial U}{\partial Y}\right)}^{2}},$$

(11) $$\beta =\frac{1}{2}\left(\frac{\partial V}{\partial X}-\frac{\partial U}{\partial Y}\right),$$

If there exists fluid rotation, there must be

(12) $$\frac{\partial V}{\partial X}{|}_{\theta }\frac{\partial U}{\partial Y}{|}_{\theta }=\alpha^{2}\sin^{2}\left(2\theta +\varphi \right)-\beta^{2}<0,$$

Thus, the angular velocity is

(13) $$\omega_{\theta ,min}=\left\{\begin{array}{c} -\beta -\alpha ,\;\;\;\;if\;\alpha^{2}-\beta^{2}<0\\ 0\;\;\;\;\;,if\;\alpha^{2}-\beta^{2}\geq 0 \end{array}\right.,$$

Where β is assumed to be negative, if β > 0, the local rotation axis can be inverted to make β negative.

Similar to the definition of vorticity, the local rotation strength is defined as

(14) $$R=2\omega_{\theta ,min},$$

## 3. Result and Discussion

### 3.1. Time-Averaged Flow Fields

**Figure 3:**
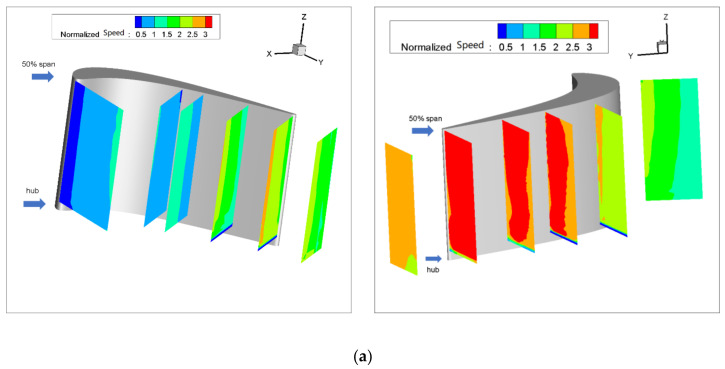

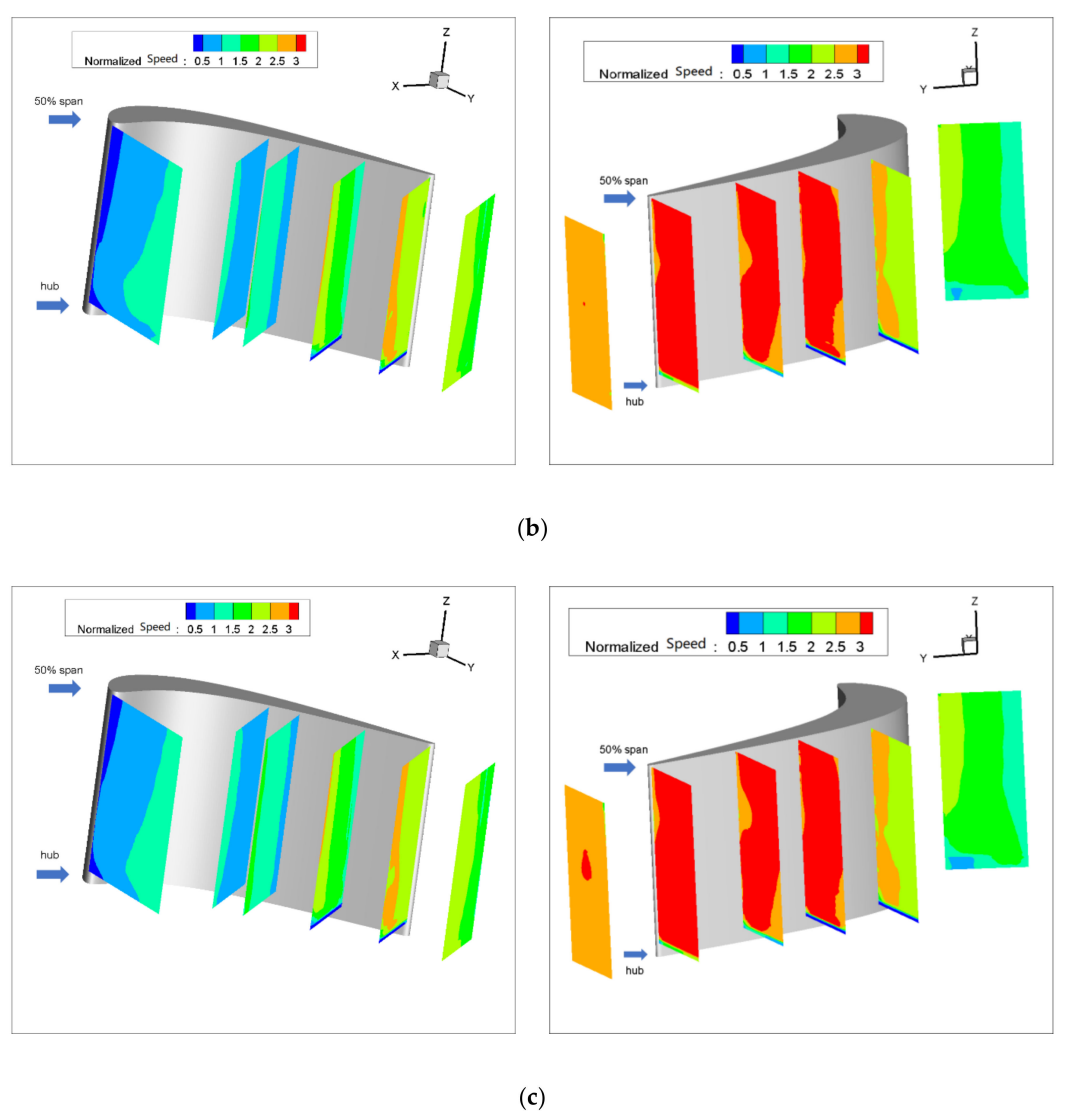
Velocity distribution: (**a**) C_m_ = 0%; (**b**) C_m_ = 1%; (**c**) C_m_ = 2%.

In this part, the time-average field of the six measurement planes will be discussed. Figure 3 demonstrates the velocity distribution of the turbine cascade passage with different purge flow rates C_m_, which is defined as

(15) $$C_{m}=\frac{{\dot{m}}_{a}}{{\dot{m}}_{s}}\times 100\%,$$

where ${\dot{m}}_{a}$ is the main flow rate and ${\dot{m}}_{s}$ is the purge flow rate.

The resultant velocity in Figure 3 is normalized by the inlet velocity of the turbine cascade. As shown in Figure 3a, for the pressure side, the flow accelerates slightly from the inlet to the exit of the cascade. The velocity distribution is linearly increasing from the pressure side to the mid passage for a specific measurement plane, while for the suction side, the flow mainly accelerates from S40 to S60 and slows down outside of the cascade. The transverse velocity distribution of the suction side is different from that of the pressure side, which is caused by the intense acceleration of the flow near the suction side. As the measurement planes are arranged several millimeters away from the suction side, the low-speed region close to the suction side is not seen from the results, due to the obstruction of the blade. When the purge flow is considered, the flow velocity in the cascade passage is generally increased, especially for the suction side. In addition, the velocity distribution of both the suction side and the pressure side is affected by the purge flow. As the circles in Figure 3 show, the low-speed zone in S80 and S100 is raised to the midspan as the purge flow increases. Furthermore, a high-speed zone appears and extends as the purge flow increases at S120. 

In order to evaluate the effect of the purge flow on the flow capacity, the blockage coefficient is defined as

(16) $$C_{b}=1-\frac{w}{\bar{w_{0,mid}}},$$

where w is the local streamwise velocity and $\bar{w_{0,mid}}$ is the mean value of the streamwise velocity at the midspan for the case without purge flow.

**Figure 4:**
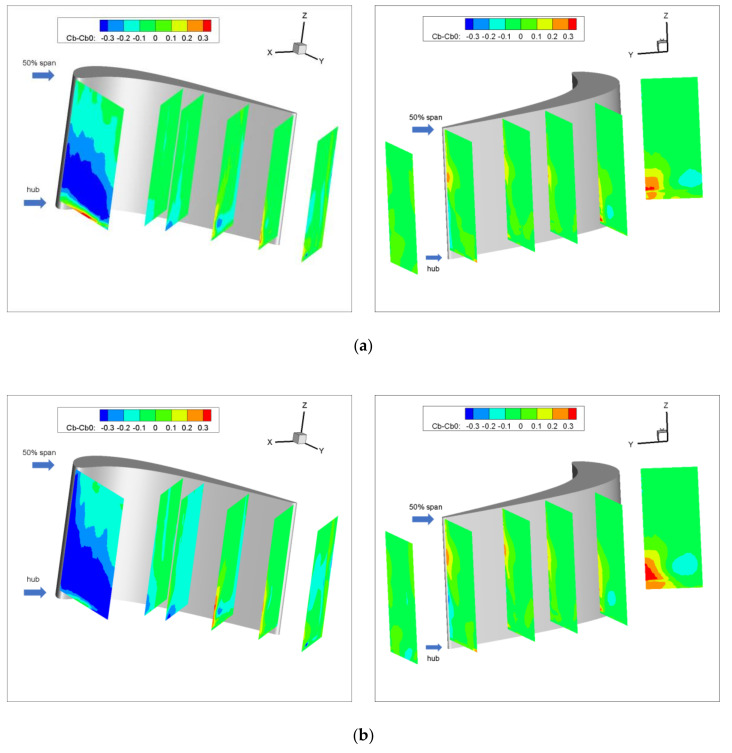
Difference in C_b_ between cases with and without purge flow: (**a**) C_m_ = 1; (**b**) C_m_ = 2.

Apparently, a higher value of the blockage coefficient means lower flow capacity. Figure 4 shows the difference in C_b_ between cases with and without purge flow. The blockage coefficient decreases at P0 for both cases with purge flow and the zone with a low blockage coefficient extends with the increase in purge flow rate, which means that the purge flow enhances the flow capacity at the pressure side of the cascade inlet. For the rest of the measurement planes at the pressure side, the blockage coefficient is merely affected by the purge flow except for some tiny areas close to the hub wall. The result of the suction side indicates that the purge flow induces a blockage region near the suction side, which migrates to the tip wall direction as the flow moves downstream. In addition, the movement of the blockage region coincides with the movement of the low-speed region in Figure 3, which should be caused by the secondary flow in the cascade passage.

**Figure 5:**
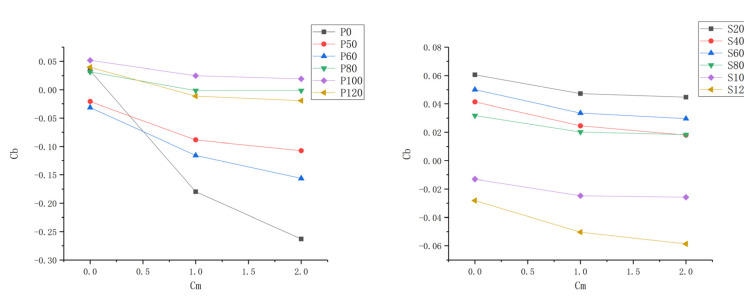
Area-weighted mean blockage coefficient of different measurement planes with three purge flows.

Figure 5 demonstrates the area-weighted mean blockage coefficient of different measurement planes with three purge flows. It can be seen that the area-weighted mean blockage coefficient decreases with the purge flow rate but with a tiny range, except for the P0 case. As P0 is closer to the rim seal, it can be concluded that the mean flow capacity of the turbine cascade is almost unaffected by the purge flow, whereas some local regions are affected by the purge flow, so may cause secondary flow loss, and will be discussed in the following part.

### 3.2. Effect of Purge Flow on Secondary Flows

**Figure 6:**
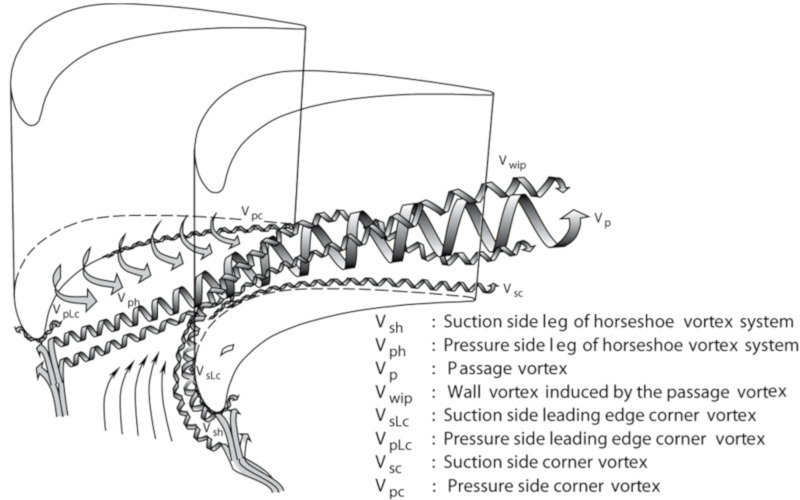
Secondary flows in a linear turbine cascade [30].

In axial turbines, secondary flow loss plays an important part in the turbine loss. Thole [29] indicates that the secondary flow loss in the first-stage vanes of a modern turbine takes up a proportion of more than 30% of the total pressure loss. Wang et al. [30] proposed a commonly used model of secondary flows in a linear turbine cascade, as shown in Figure 6. Both the horseshoe vortex and the passage vortex are in contact with the boundary layer of the hub wall, while the purge flow inevitably interacts with the boundary layer, which will affect the secondary flows in the turbine cascade. In this part, the effect of purge flow on the secondary flow will be discussed.

In order to investigate the effect of purge flow on secondary flows, vortex identification with the Rortex method is performed on all the measurement planes. In addition, the velocity vectors are also shown in the following figures. The result indicates that there is almost no difference in the vortex distribution for P50 and P60. For the result of P80, P100, and P120, the affected regions are far away from the pressure side, which is caused by the passage vortex near the suction side. Therefore, except for P0, the results of the pressure side will not be discussed in this paper, for brevity. 

**Figure 7:**
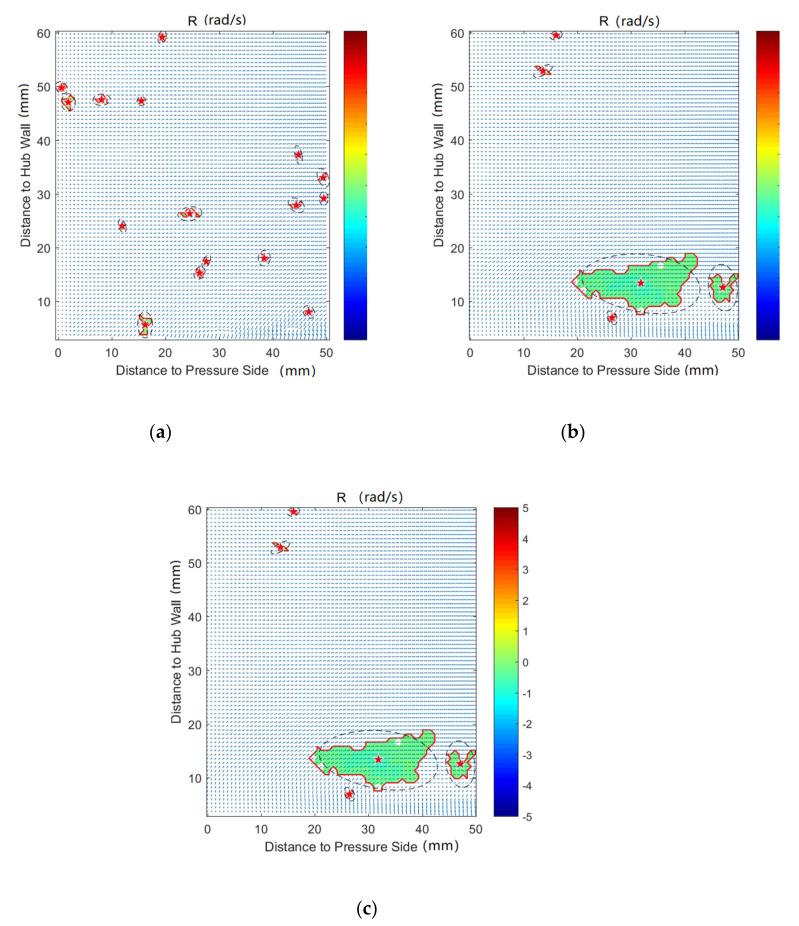
Vortex distribution at P0: (**a**) Cm = 0; (**b**) Cm = 1; (**c**) Cm = 2.

Figure 7 shows the vortex distribution of P0, in which the vortex border and vortex core are marked with a red line and red star, respectively. The black dashed line is the result of ellipse fitting on the vortices. For the case without purge flow, there is no concentrated vortex in the whole plane. Combined with Figure 3a, it can be concluded that the flow field of P0 is fairly uniform without purge flow. For the cases of Cm = 1% and Cm = 2%, vortices appear near the hub wall region. According to Figure 2, these regions are close to the rim seal and far away from the blade leading edge. Therefore, the vortices in P0 should be induced by the interaction of the main flow and the purge flow. Rabs et al. [31] identified the Kelvin–Helmholtz vortices near the rim cavity, which is caused by the shear of the main flow and purge flow. The vortices in P0 should be the result of the downstream propagation of the Kelvin–Helmholtz vortices, which can also be proved by the low uniform local vortex strength in these regions.

**Figure 8:**
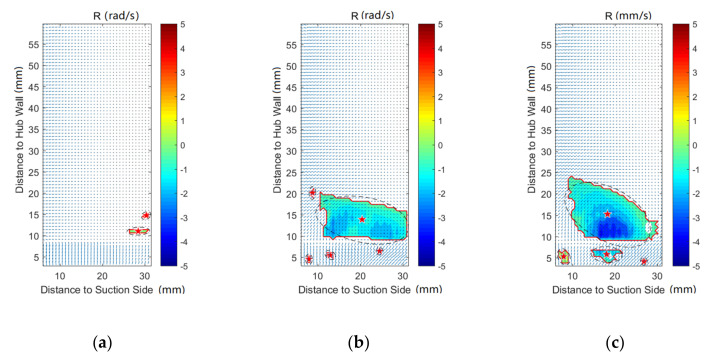
Vortex distribution at S20: (**a**) Cm = 0; (**b**) Cm = 1%; (**c**) Cm = 2%.

**Figure 9:**
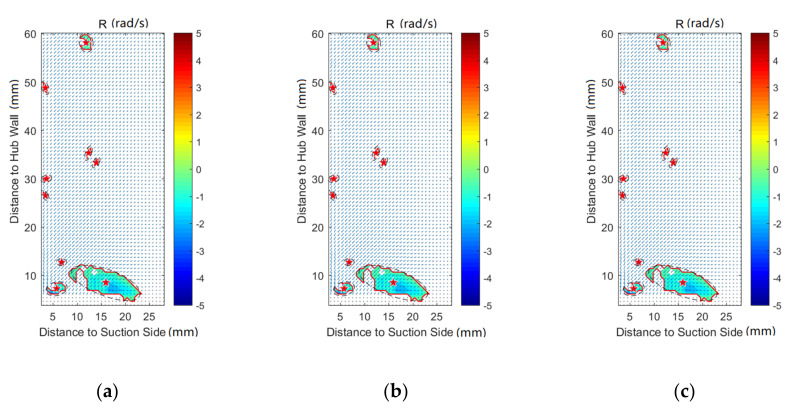
Vortex distribution at S40: (**a**) Cm = 0; (**b**) Cm = 1%; (**c**) Cm = 2%.

**Figure 10:**
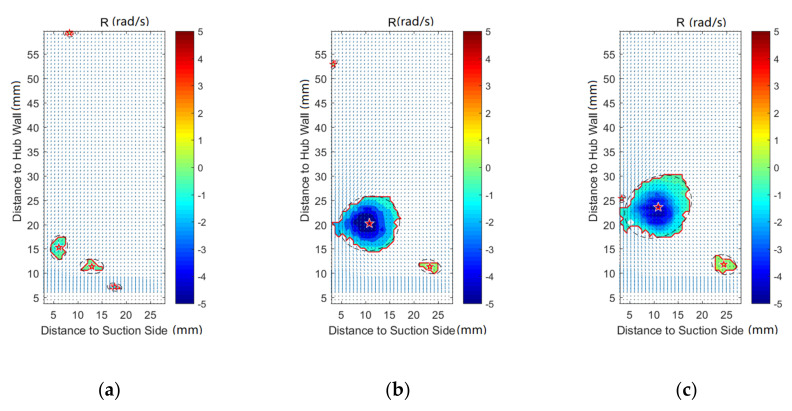
Vortex distribution at S60: (**a**) Cm = 0; (**b**) Cm = 1%; (**c**) Cm = 2%.

**Figure 11:**
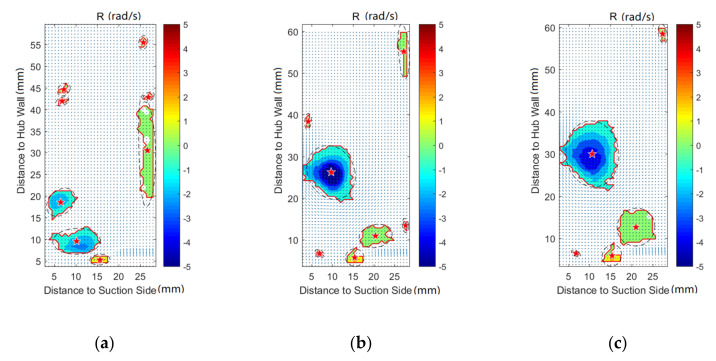
Vortex distribution at S80: (**a**) Cm = 0 (**b**) Cm = 1%; (**c**) Cm = 2%.

**Figure 12:**
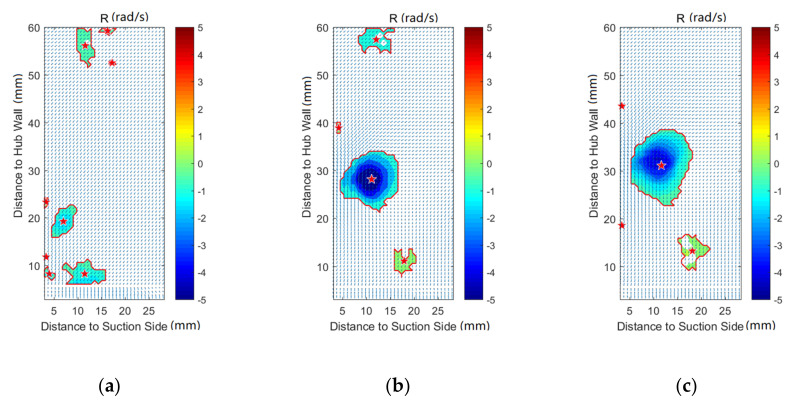
Vortex distribution at S100: (**a**) Cm = 0; (**b**) Cm = 1%; (**c**) Cm = 2%.

**Figure 13:**
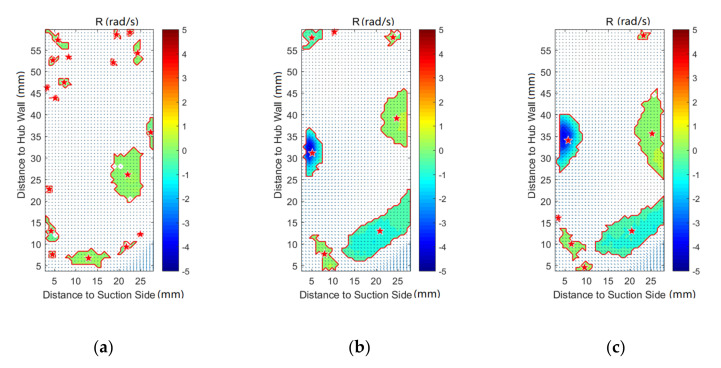
Vortex distribution at S120: (**a**) Cm = 0; (**b**) Cm = 1%; (**c**) Cm = 2%.

Figure 8, Figure 9, Figure 10, Figure 11, Figure 12 and Figure 13 demonstrate the results of Rortex identification of the suction side for three purge flow rates. When there is no purge flow, no remarkable vortex structures are observed at S20. As the flow develops downstream, some low-strength vortices emerge in the low-span area near the suction side. As is shown in Figure 13a, when the fluid flows out of the cascade, lots of small-sized vortices with lower strength show up, which means the stability of the flow field falls rapidly outside the passage.

For all the cases with purge flow, a concentrated vortex appears near the hub wall, the core of which is marked with a red star. The deviation between the position of the recognized vortex core and the actual value in Figure 8 is caused by the PIV measurement error, which arises from the reflection of the hub wall. According to the velocity vectors in these following figures, the rotation direction of the identified vortex is the same as that of the passage vortex V_p_ shown in Figure 6. Through comparison of Figure 8, Figure 9, Figure 10, Figure 11, Figure 12 and Figure 13, it is obvious that the identified vortex migrates upwards as the flow develops downstream, which is the same as the motion form of the passage vortex. Combined with the position of the vortex, it can be inferred that the vortex in Figure 8, Figure 9, Figure 10, Figure 11, Figure 12 and Figure 13 is the passage vortex that appears earlier due to the interaction of purge flow and main flow. The passage vortex moves upward as the purge flow rate increases. As shown in Figure 9, Figure 10, Figure 11, Figure 12 and Figure 13, a vortex with lower strength is induced by the passage vortex. The rotating direction of the induced vortex is opposite to the passage vortex. In addition, the size of the induced vortex increases with the purge flow rate and the downstream development of the flow. In conclusion, the secondary flow of the turbine cascade is strengthened by the purge flow, and larger purge flow rate means stronger secondary flow, which means the secondary flow loss could also be increased by the purge flow.

**Figure 14:**
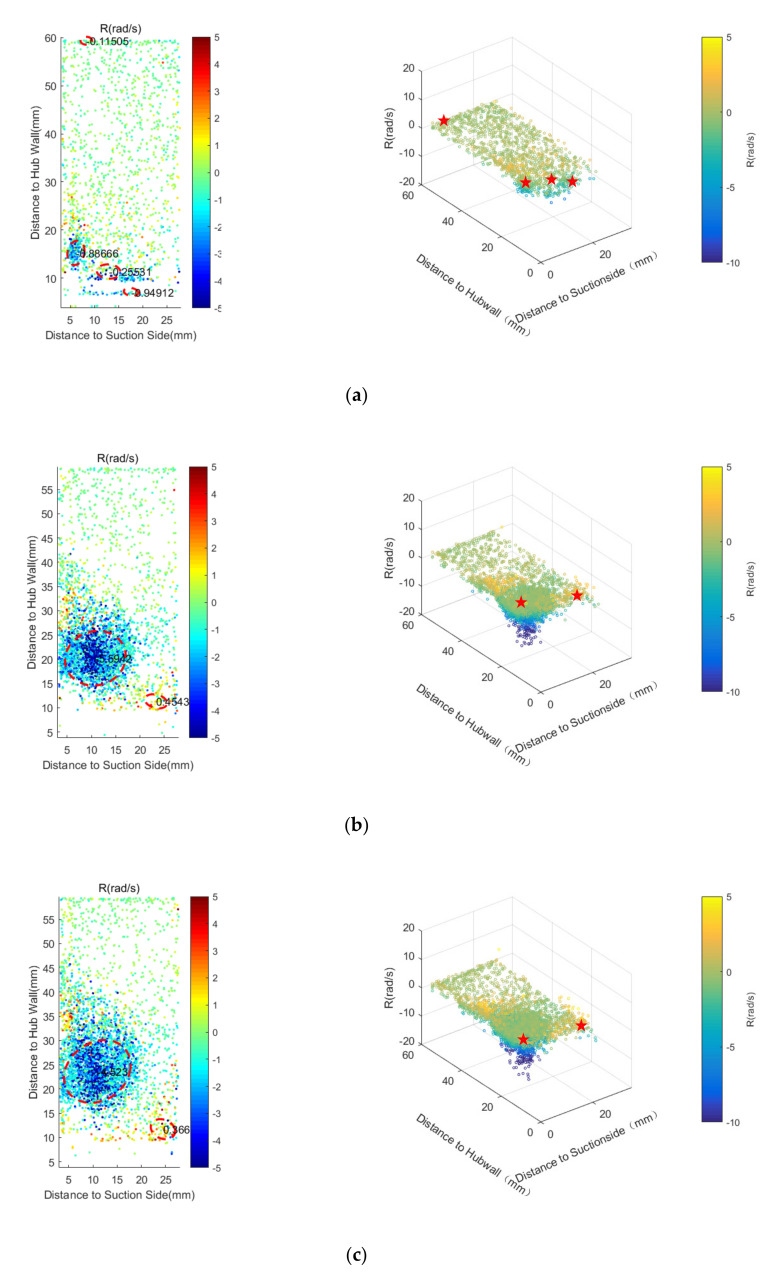
Statistical result of the instantaneous R for S60: (**a**) Cm = 0; (**b**) Cm = 1%; (**c**) Cm = 2%.

In order to study the effect of purge flow on the instantaneous flow field, the Rortex identification method was adopted to each instantaneous PIV result of S60. Figure 14 shows the statistical result of the vortex identification. The colored dots in Figure 14 mark the identified vortex core of all the instantaneous results, whose color represents the R value of the vortex core. The red circles with the dashed lines in the left three images are the result of ellipse fitting on the time-averaged vortices. The black numbers in the circles are the R values of the time-averaged vortex core. The red stars in the right images are the vortex cores of the time-averaged results. It is obvious that even for the case without purge flow, there are low-strength vortices in the whole measurement plane. The density of the instantaneous vortices increases as they get closer to the time-averaged vortex cores, which is particularly evident for the cases with purge flow. For the cases with purge flow, the area affected by the time-averaged passage vortex is apparently smaller than the vortices-intensive area in the statistical results. In addition, as shown in the right images, the strength of some instantaneous vortices is larger than that of the time-averaged vortices. Therefore, the time-averaged result underestimates the effect of vortices no matter the range of influence or the strength. 

**Figure 15:**
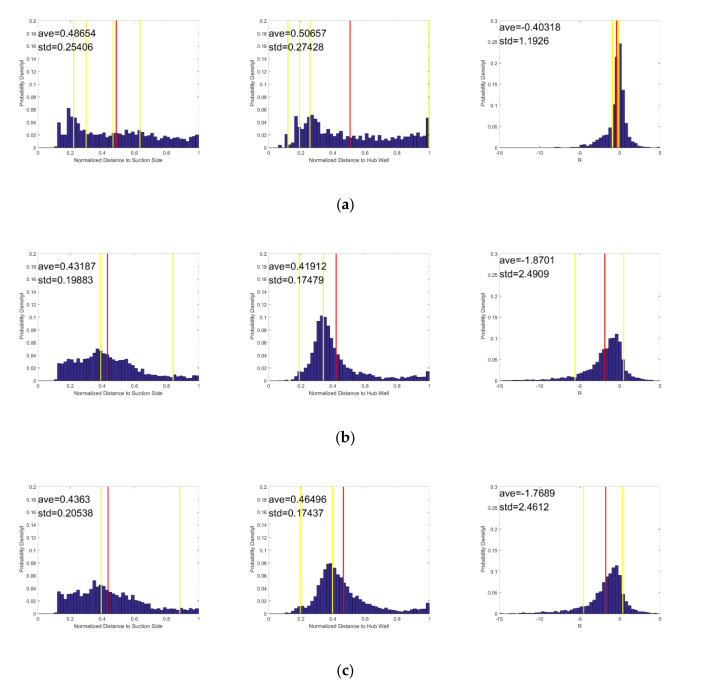
Probability density distribution of normalized distance to the suction side, normalized distance to the hub wall, and the R value of the instantaneous vortex core at S60. The red line is the mean result of the instantaneous vortices, and the yellow line is the result of the time-averaged vortices. (**a**) Cm = 0; (**b**) Cm = 1%; (**c**) Cm = 2%.

Figure 15 shows the probability density distribution of the instantaneous vortices’ normalized distance to the suction side, normalized distance to the hub wall, and the R value. The yellow line represents the time-averaged vortices, and the red line is the mean result of the instantaneous vortices. The mean value and the standard deviation are also displayed in the images. As shown in Figure 15, the peak values of the probability density distribution correspond to the time-averaged vortices. However, for the cases with purge flow, the corresponding peak values of the weaker vortices are not prominent. 

As Figure 15b,c show, the peak values of the normalized distance to the hub wall moves to the positive direction of the x-axis, while the peak values of the normalized distance to the suction side are hardly affected by the purge flow rate. The variations in the peak vales indicate that the passage vortex migrates upward in the span-wise direction and keeps stable in the pitch-wise direction as the purge flow rate increases, which can also be seen in Figure 10. 

The standard deviation of the normalized distance to the suction side and the normalized distance to the hub wall decreases when the purge flow is introduced, which indicates that the purge flow makes the vortices more concentrated. As the purge flow rate increases from 1% to 2%, the standard deviation of the normalized distance to the suction side and the normalized distance to the hub wall hardly changes, and so do the mean value and the standard deviation of the R value. Therefore, it can be concluded that the passage vortex decreases the sensitivity of the main flow to purge flow, making the flow field more stable. 

### 3.3. Entropy Production with Different Purge Flow Rates

As the experiment facility is a low-speed turbine cascade, the flow in the cascade can be regarded as isothermal, so the gradients and the fluctuation in temperature can be ignored. Thus, Equation (4) is adopted to investigate the entropy generation due to flow loss caused by purge flow in this part.

**Figure 16:**
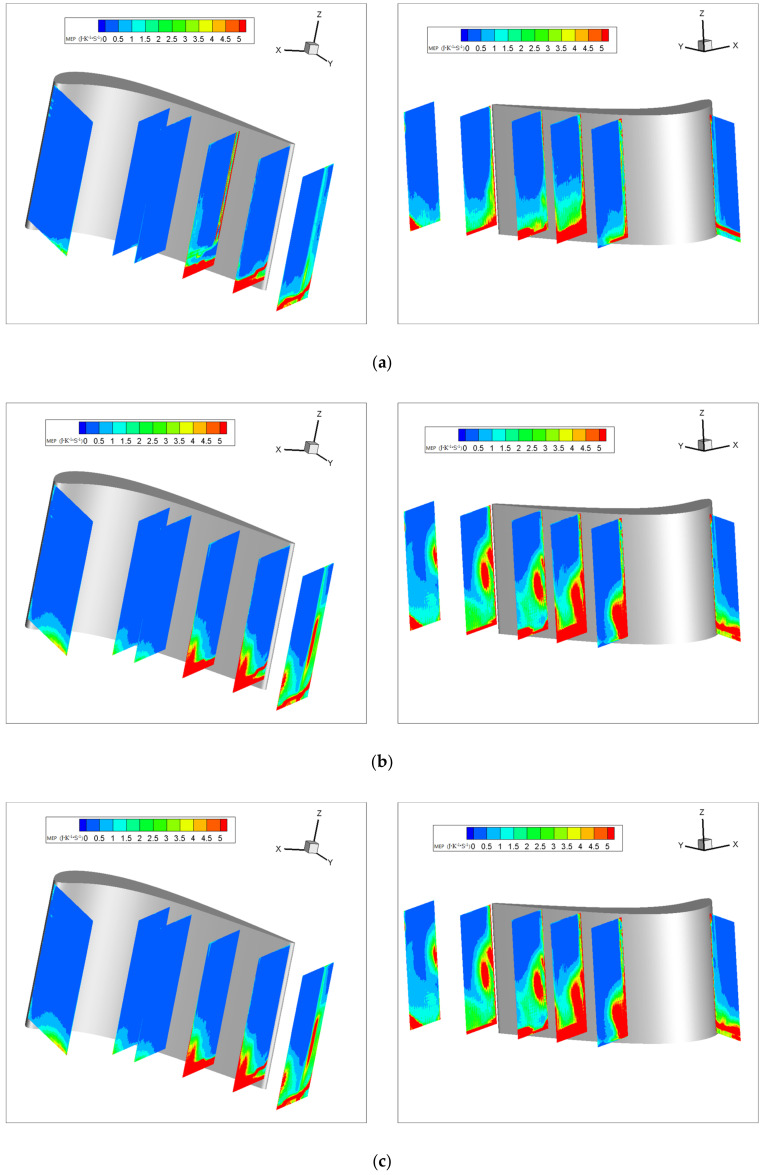
Mean entropy production: (**a**) Cm = 0; (**b**) Cm = 1%; (**c**) Cm = 2%.

Figure 16 shows the mean entropy production (MEP) for different purge flow rates. For both the suction side and the pressure side, the MEP increases with the purge flow rates. However, for the first few measurement planes of the pressure side, including P0, P50, and P60, the regions of increasing MEP are concentrated in the bottom left of the measurement planes, which coincides with the blockage area in Figure 4. For P80 and P100, the high-MEP area caused by purge flow is larger than those of the former ones. Apparently, the high MEP is caused by the passage vortex. The MEP of P120 is affected by both the passage vortex and the wake of the blade, as shown in the bottom left and the right side of P120. For the suction side, the high-MEP region corresponds to the region with high R values in Figure 8, Figure 9, Figure 10, Figure 11, Figure 12 and Figure 13. In addition, the high-MEP region also migrates to the tip wall direction as the flow develops downstream and the purge flow increases, which is exactly the same as the passage vortex. 

Therefore, it can be concluded that the entropy caused by secondary flow loss is caused by the blockage effect and the passage vortex. The blockage-effect-caused entropy production is concentrated on the fore of the pressure side, where the passage vortex has not appeared. The vortex-caused entropy production appears near the suction side and downstream of the pressure side. Furthermore, the vortex-caused entropy production is much larger than the blockage-effect-caused entropy.

**Figure 17:**
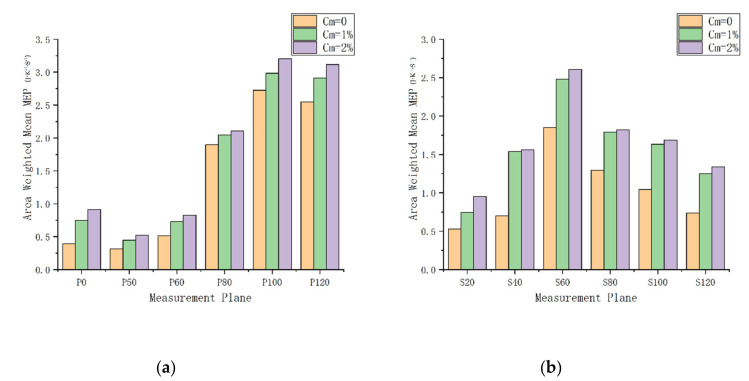
Area-weighted mean entropy production (MEP): (**a**) Pressure side; (**b**) suction side.

Figure 17 shows the area-weighted mean MEP. In order to eliminate the incorrect results caused by the hub wall reflection, some of the results near the hub wall are ignored when calculating the area-weighted mean MEP. For all the measurement planes, the area-weighted mean MEP increases with the purge flow rates due to the interaction of the main flow with the purge flow. For the pressure side, the area-weighted mean MEP generally increases as the flow develops downstream. However, there is a sudden increase in the area-weighted mean MEP in P80 due to the passage vortex, which was discussed in Section 3.2, while for the suction side, the area-weighted mean MEP reaches a maximum at S60. This should be caused by the development of the passage vortex close to the suction side. The blade profile shown in Figure 1 indicates that the fluid accelerates and turns in the anterior of the cascade passage, causing a large pressure difference between the pressure side and suction side, which induces the passage vortex. The passage vortex originating from the blade leading edge develops and reaches a maximum at about S60. When moving downstream at S60, the passage vortex does not intensify any more, as the flow no longer accelerates and turns. Therefore, the area-weighted mean MEP caused by the secondary flow loss peaks at S60. In addition, the area-weighted mean MEP increases with the purge flow rate, while at S40 and downstream, where the passage vortex fully develops, the difference in area-weighted MEP between the case of Cm = 1% and Cm = 2% is smaller than others. This means that the passage vortex suppresses the sensitivity of the area-weighted MEP to the purge flow rate. 

## 4. Conclusions

The influence of interaction between main flow and purge flow was investigated experimentally with the stereoscopic PIV technique. The Rortex method was adopted to study the effect of purge on the secondary flow in the cascade. In addition, a mean entropy production equation was utilized to evaluate the secondary flow loss. The following conclusions can be derived from this paper:

(1) The purge flow basically increases the flow velocity in the cascade passage, especially for the suction side. In the meantime, the purge flow induces a blockage region near the suction side, which migrates to the tip wall direction as the flow develops downstream. In addition, the blockage area also moves upward with the increasing purge flow rate.

(2) The purge flow has a prominent impact on both the generation and the motion of the passage vortex. The purge flow promotes the generation of the passage vortex near the suction side. Furthermore, the passage vortex migrates to the tip wall direction with increasing purge flow rate, enlarging the region affected by the secondary flow.

(3) The time-averaged results understate the effect of the instantaneous vortices in both the range of influence and the strength. The statistical results show that the passage vortex induced by the purge flow reduces the sensitivity of the flow field to the purge flow rate.

(4) The mean entropy production (MEP) shows that the flow loss is mainly caused by the passage vortex. The coincidence of the high-MEP region and the location of the passage vortex indicates that the purge flow increases the secondary flow loss by affecting the generation and development of the passage vortex.

## Figures and Tables

**Table 1:** Parameters of the Turbine Cascade.

Number of Blades	5
Chord Length	102 mm
Axial Chord	71 mm
Span	120 mm
Pitch	93.75 mm
Inlet Flow Angle	33.6°
Outlet Flow Angle	67°

## Nomenclature

Cb	Blockage Coefficient
Cm	Non-dimensional Purge Flow Rate
Cw, min	Non-dimensional Minimum Sealing Flow Rate to Prevent Gas Ingestion
Cp, max	Non-dimensional Pressure Coefficient
k	Thermal Conductivity, W/(m·K)
${\dot{m}}_{a}$	Main Flow Rate, kg/s
${\dot{m}}_{s}$	Purge Flow Rate, kg/s
${\dot{P}}_{s}$	Entropy Production Rate, J/(KS)
Q	Transfer Matrix
$Q^{T}$	Transpose of Matrix Q
r	Rotating Axis
R	Rortex Value, rad/s
u, v	Velocity in xyz Frame, m/s
U, V	Velocity in XYZ Frame, m/s
∇V	Tensor of Velocity Gradient
$\tau_{ij}$	Viscous Stress Arising from Velocity Gradients in the Fluid Motion
μ	Dynamic Viscosity, N·s/㎡
δij	Kronecker delta
θ	Rotating Angle, rad
$\omega_{\theta }$	Angular Velocity, rad/s
